# Supplementary material for: ProteinShader: illustrative rendering of macromolecules
Source: BMC Struct Biol. 2009 Mar 30;9:19. doi: 10.1186/1472-6807-9-19 (PMC2672931; doi:10.1186/1472-6807-9-19)
Supplement: Additional file 1 — ProteinShader program without source code. This compressed file contains the complete ProteinShader program including associated libraries, but no source code. A README.txt file gives an overview of the ProteinShader distribution, and the index.html file in the help subdirectory has directions on getting started with the program as well as a set of tutorials. [file 1472-6807-9-19-S1.zip › ProteinShader-beta-0_9_4-binary/help/api/org/proteinshader/graphics/typography/package-summary.html]

org.proteinshader.graphics.typography (ProteinShader API)


|  |  |  |
| --- | --- | --- |
| **Overview** \| **Package** \| Class \| **Use** \| **Tree** \| **Deprecated** \| **Index** \| **Help** | |  |
| **PREV PACKAGE**   **NEXT PACKAGE** | **FRAMES**    **NO FRAMES**     **All Classes** |  |


---

## Package org.proteinshader.graphics.typography

Holds the classes needed for applying text labels to the curved surfaces of
tubes and ribbons.

**See:**
  
          **Description**

| **Interface Summary** | |
| --- | --- |
| **GlyphSetFactory** | Defines the interface for a factory that can read glyphs from a source and produce a GlyphSet object. |

| **Class Summary** | |
| --- | --- |
| **GLF2GlyphSetFactory** | Reads a set of glyphs from a ".glf" file that was created with the glFont program (Version 2.0) written by Brad Fish (brad.fish@gmail.com). |
| **GLF2Header** | Stores information from the header of a '.glf' file. |
| **Glyph** | Stores a 2D array of bytes that form a graphical representation of a character. |
| **GlyphDemo** | Provides a driver to perform some basic tests on classes in the typography package. |
| **GlyphSet** | Stores a set of Glyph objects. |
| **GlyphSetFactoryFactory** | Use Java reflection to load a concrete GlyphSetFactory based on information held in a Properties object. |
| **TextLabel** | Stores a byte buffer with the bitmap for a text label that can be applied to the curved surface of a 3D tube or ribbon. |
| **TextLabelFactory** | Uses a GlyphSet to create short text labels to place on segments of a tube or ribbon. |
| **TextLabelManager** | Knows how to create and cache OpenGL display lists holding the glTexSubImage() with a bitmap for a text label that can be pasted onto curved surfaces. |

## Package org.proteinshader.graphics.typography Description

Holds the classes needed for applying text labels to the curved surfaces of
tubes and ribbons.
